# The Development of the Stress-Free Polishing System Based on the Positioning Error Analysis for the Deterministic Polishing of Jet Electrochemical Machining

**DOI:** 10.3390/mi15030393

**Published:** 2024-03-14

**Authors:** Ke Wang, Hongding Wang, Yanlong Zhang, Huirong Shi, Jiahao Shi

**Affiliations:** 1 School of Mechanical Engineering, Lanzhou Jiaotong University, Lanzhou 730070, China; wanghongding@gmail.com (H.W.); zhangyl@mail.lzjtu.cn (Y.Z.); shrz98@aliyun.com (H.S.); 2 Department of Bioresource Engineering, McGill University, Montreal, QC H9X 3V9, Canada; jiahao.shi@mcgill.ca

**Keywords:** stress-free polishing, deterministic polishing, jet electrochemical machining, positioning accuracy, surface flatness

## Abstract

Deterministic polishing based on jet electrochemical machining (Jet-ECM) is a stress-free machining method for low-rigidity and ultra-precision workpieces. The nozzle is equivalent to a special tool in deterministic polishing, and the workpiece material is removed using the mechanism of electrochemical dissolution at the position where the nozzle passes. By precisely regulating the nozzle’s movement speed and dwell time, the quantity of material removed from the workpiece at a designated position can be finely adjusted. With this mechanism, the improvement of the workpiece shape accuracy can be achieved by planning the nozzle trajectory and nozzle movement speed. However, due to the positioning errors of the polishing device, the actual position of the nozzle may deviate from the theoretical position, resulting in errors in material removal amount, which affects the accuracy and stability of the polishing process. This study established a mathematical model to analyze the influence of nozzle positioning errors in deterministic polishing based on Jet-ECM. This model has been used to design a specific deterministic polishing device based on Jet-ECM. With the proposed deterministic polishing device, the surface shape of the workpiece is converged. The surface peak-to-valley (PV) value of the φ 50 mm workpiece (valid dimensions = 90% of the central region) indicated that the shape error of the surface was reduced from 2.67 μm to 1.24 μm in 34 min. The power spectral density (PSD) method was used to evaluate the height distribution and height characteristics of the workpiece surface. The results show that the low frequency spatial error is reduced significantly after processing. This study improves the accuracy of the stress-free deterministic polishing methods and further expands the use of deterministic polishing in industry.

## 1. Introduction

Copper (Cu) surfaces with high flatness are widely used as substrates for functional materials [1], high-power laser devices [2], and precision physics experiments [3]. The surface shape accuracy of Cu directly affects the performance of the devices. Therefore, preparing high-flatness Cu surfaces can provide a reliable guarantee for the use of subsequent devices [4].

To obtain an ultra-flat Cu surface, the most commonly used processing method is chemical mechanical polishing (CMP). CMP achieves material removal through synergistic chemical and mechanical effects. Through chemical effects, the mechanical force during the process can be reduced significantly. However, it still requires mechanical force to achieve material removal, which leads to the deformations of the polishing pad and workpiece [5,6,7]. To further improve the flatness of the workpiece, deterministic machining, which utilizes computer control technology, has emerged. The most commonly used deterministic machining method is small tool grinding. By controlling the moving path and dwell time of the small tool, the material removal amounts at different positions of the workpiece can be controlled. The rapid improvement of the workpiece shape accuracy is achieved. The surface measurements and processing processes are repeated until the workpiece shape accuracy achieves the target shape accuracy [8,9,10]. However, for low-rigidity and ultra-precision workpieces, both the clamping deformation and stress deformation lead to workpiece deformation. Even if the workpiece obtains a high surface accuracy after processing, the workpiece will rebound after discharge from the clamping, which limits further improvement of the shape accuracy [11,12]. Therefore, stress-free deterministic processing is the key to solving this problem, which does not need mechanical force and clamping during the processing process.

Jet electrochemical machining (Jet-ECM) is an unconventional stress-free machining method that relies on anode dissolution to achieve material removal. This method has the advantages of high removal efficiency, no cathode consumption, no heat-affected zone, and no residual stress, which is commonly recognized as a stress-free polishing method [13,14,15,16,17]. Zhou and Wang et al. (2020) [18,19] proposed a new stress-free deterministic polishing method based on the principle of computer-controlled optical surfacing (CCOS) and Jet-ECM. During the stress-free deterministic polishing, the nozzle (cathode) is equivalent to a special tool. At the workpiece position where the nozzle passes, the workpiece material is removed by the effects of the anode dissolution. Furthermore, combined with path planning and nozzle speed control, surface accuracy improvement can be achieved. This specific stress-free deterministic polishing was used for the flattening of the Cu workpiece. After processing two times, an ultra-flat Cu surface with a surface peak and valley (PV) of 1.7 μm was obtained. However, existing research showed that the positioning errors of the tool have a significant impact on the processing process. Liang et al. (2018) [20] proposed that the positioning errors caused by installation would lead to deviations between the predicted and the actual surface. The author used an on-machine measurement method to reduce the installation errors of the tool. The accuracy and efficiency were improved. Sun et al. (2020) [21] designed a deterministic machining device based on finite element analysis, which was used for achieving deterministic machining of ultra-precision shaft parts. However, the specific stress-free deterministic polishing based on Jet-ECM has characteristics. In Jet-ECM, the corrosive electrolyte flow is shot from the nozzle to the workpiece surface at a very high speed. Additionally, the workpiece material removal can be limited to the central area of the jet to form a Gaussian-type distribution. Saxena et al. (2020) [22] developed a prototype precision hybrid laser-ECM machine device that consists of a granite gantry-type machine frame, a pulsed laser source, an electrolyte supply system, a tool holding system, a control unit, and a machining cell for the hybrid laser-electrochemical processing. Liu et al. (2021) [23] investigated the effects of an inclined nozzle and the nozzle movement direction on the shape and surface roughness in Jet-ECM. The results showed that the groove shape and surface roughness can be affected by the movement direction with a certain nozzle inclination angle. However, the influence of nozzle positioning errors on the machining accuracy in deterministic polishing based on Jet-ECM has not been studied. Therefore, studying the influence of nozzle positioning errors on machining accuracy is of great help in understanding the mechanism, and guiding the equipment design.

The specific deterministic machining method and the algorithm used to determine the parameters of the deterministic polishing based on Jet-ECM were proposed in 2020 [18,19]. In 2022 [24], the process parameters of the Jet-ECM, which influence the accuracy and surface quality, were analyzed to optimize the process parameters of Jet-ECM. The optimized parameters of Jet-ECM are beneficial to improve the accuracy and stability of the polishing. Continuously, in this study, a mathematical model was established to analyze the influence of nozzle positioning errors on the specific stress-free deterministic polishing. Based on the analysis results, a machining device used in the deterministic polishing based on Jet-ECM was designed. This device can meet the requirements of the machining. Finally, an ultra-flat Cu workpiece was processed with the proposed polishing device.

## 2. Mechanism

### 2.1. The Mechanism of Jet-ECM

**Figure 1:**
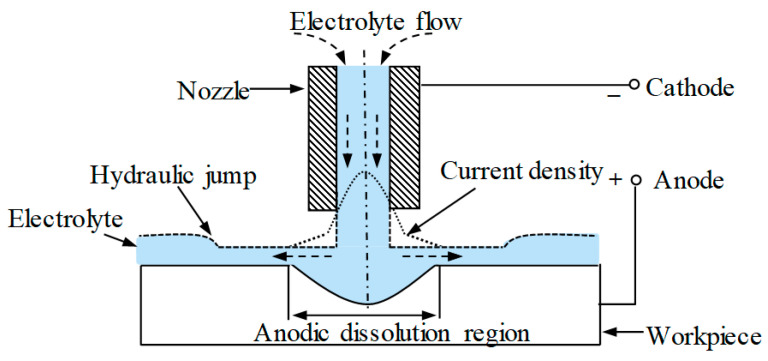
A schematic of the Jet-ECM.

In Jet-ECM, the material removal is achieved by the mechanism of electrochemical dissolution. As is shown in Figure 1, the workpiece is connected to the anode of the power, and the nozzle is connected to the cathode. There is a small gap between the nozzle and the workpiece to discharge the electrolyte flow. When the electrolyte flow hits the workpiece surface, the electrolyte flow speeds and electrolyte flow directions change drastically. A thin electrolyte flow layer is, thus, formed. The thin layer is generated around the jet center and the thickness of the layer rises suddenly at the edge of the thin layer to form a hydraulic jump phenomenon. With the help of the hydraulic jump phenomenon, the thin layer-covered region has high resistance [25], and most of the currents are restrained in this region. Far away from the jet center, both the current density and the material removal rate (MRR) are decreased to form a Gaussian-type pit on the surface [26].

At the anode (workpiece), metal atoms (N) dissolve into ions, accompanied by oxygen generation [27]:

(1) $${N+2H}_{2}O\to N^{z+}{+O}_{2}↑{+4H}^{+}+(z{+4)e}^{-}$$

where *z* represents the number of electrons released during the metal dissolution reaction.

At the cathode (nozzle), the water molecules in the electrolyte decompose into hydroxyl ions and hydrogen gas, according to Equation (2):

(2) $${2H}_{2}{O+2e}^{-}\to {2\mathrm{OH}}^{-}{+H}_{2}↑$$

### 2.2. The Mechanism of Deterministic Polishing Based on Jet-ECM

**Figure 2:**
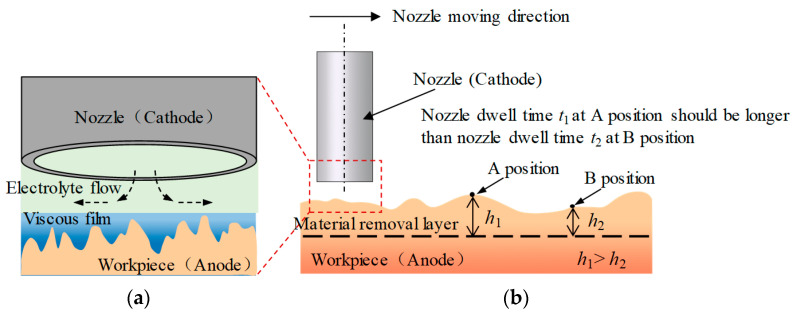
A deterministic polishing method based on Jet-ECM (**a**) The enlarged view (**b**) The schematic diagram.

In deterministic polishing based on Jet-ECM, the nozzle (cathode) becomes a special tool. The material removal occurs when the nozzle passes. By controlling the nozzle moving speeds and nozzle dwell time, the material removal amounts of the workpiece can be controlled. By increasing the dwell time of the nozzle at the convex points of the workpiece, the material removal amounts at the convex point increase. Based on this mechanism, combined with the nozzle path planning, the workpiece can be flattened, as is shown in Figure 2.

In this study, the nozzle adopts a circular path. During the processing process, the nozzle revolves at the specified movement speeds and radiuses around the symmetric center of the workpiece to generate different ring grooves. The nozzle passes through the convex area of the workpiece at a low speed to increase the dwell time. The nozzle sweeps through the concave area at a high speed, reducing the dwell time and the material removal amounts. In this way, a flat surface can be achieved. The optimal nozzle traveling path and moving speeds can be calculated using the mathematical model proposed before [18,19].

### 2.3. The Influence of Nozzle Positioning Errors on Deterministic Polishing Based on Jet-ECM

**Figure 3:**
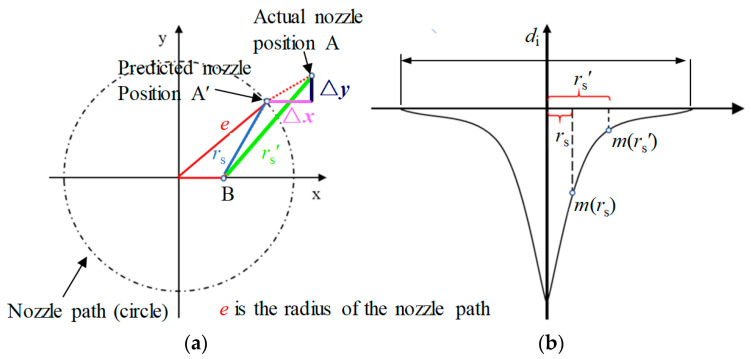
The influence of positioning errors on stress-free deterministic polishing. (**a**) The path of the nozzle is a circle. (**b**) A schematic diagram of the MRR profile.

Based on the model [18,19], the machining parameters, such as nozzle traveling path and movement speeds can be calculated by comparing the initial and target surface shape of the workpiece. However, because of the installation errors and limitations in device accuracy, nozzle positioning errors arise between the actual nozzle positions and the theoretical positions. Assume that there are nozzle positioning errors (Δ*x*, Δ*y*) between the theoretical and actual positions in the X and Y directions, respectively. The positioning errors cause a distance deviation between the actual distance *r*_s_′ (A′ B) and theoretical distance *r*_s_ (AB) (Figure 3a) and the MRR deviation *ε* is caused. As the distance deviation between *r*_s_′ and *r*_s_ increases, the *ε* increases (Figure 3b):

(3) $$\varepsilon ({r_{s}}^{\prime }-r_{s})=\left|m({r_{s}}^{\prime })-m(r_{s})\right|$$

where *r*_s_ is the distance between an arbitrary theoretical nozzle position A and a random location B on the workpiece surface, and *r*_s_′ is the distance between the actual nozzle position A′ and a random location B. The material removal function *m*(*r*_s_) is a mathematical function to describe the line profile of the Jet-ECM MRR.

As is shown in Figure 3a.

(4) $${r_{s}}^{\prime }-r_{s}<\sqrt{\Delta x^{2}+\Delta y^{2}}$$

Combining Equations (3) and (4), we can obtain:

(5) $$\varepsilon ({r_{s}}^{\prime }-r_{s})<\varepsilon (\sqrt{\Delta x^{2}+\Delta y^{2}})$$

(6) $$\varepsilon (\sqrt{\Delta x^{2}+\Delta y^{2}})=\left|m(r_{s}+\sqrt{\Delta x^{2}+\Delta y^{2}})-m(r_{s})\right|$$

combining Equations (3), (5) and (6), we can obtain:

(7) $$\left|m({r_{s}}^{\prime })-m(r_{s})\right|<\left|m(r_{s}+\sqrt{\Delta x^{2}+\Delta y^{2}})-m(r_{s})\right|$$

*k*_w_ is set as the deviation rate, which represents the influence of nozzle positioning errors (Δ*x*, Δ*y*) on the specific stress-free deterministic polishing. In addition, *k*_w_ should be less than 5%:

(8) $$k_{w}=\frac{\left|m({r_{s}}^{\prime })-m(r_{s})\right|}{m(r_{s})}$$

combining Equations (7) and (8), we can obtain:

(9) $$\frac{\left|m({r_{s}}^{\prime })-m(r_{s})\right|}{m(r_{s})}<\frac{\left|m(r_{s}+\sqrt{\Delta x^{2}+\Delta y^{2}})-m(r_{s})\right|}{m(r_{s})}$$

to simplify, it is assumed that *m*(*r*_s_) is as follows:

(10) $$m(r_{s})=Ae^{-\frac{{r_{s}}^{2}}{d^{2}}}$$

where *d* is the eigenvalue of the Gaussian function *m*(*r*_s_):

(11) $$m(r_{s}+\sqrt{\Delta x^{2}+\Delta y^{2}})=Ae^{-\frac{{\left(r_{s}+\sqrt{\Delta x^{2}+\Delta y^{2}}\right)}^{2}}{d^{2}}}$$

the *k*_w_ is derived as:

(12) $$k_{w}<\left|e^{\frac{{r_{s}}^{2}-{\left(r_{s}+\sqrt{\Delta x^{2}+\Delta y^{2}}\right)}^{2}}{d^{2}}}-1\right|$$

(13) $${\left(r_{s}+\sqrt{\Delta x^{2}+\Delta y^{2}}\right)}^{2}\geq {r_{s}}^{2}+{\left(\sqrt{\Delta x^{2}+\Delta y^{2}}\right)}^{2}$$

combining Equations (12) and (13), we can obtain:

(14) $$k_{w}\leq \left|e^{\frac{{r_{s}}^{2}-({r_{s}}^{2}+(\sqrt{\Delta x^{2}+\Delta y^{2}}{)}^{2})}{d^{2}}}-1\right|$$

(15) $$\left|e^{\frac{{r_{s}}^{2}-({r_{s}}^{2}+(\sqrt{\Delta x^{2}+\Delta y^{2}}{)}^{2})}{d^{2}}}-1\right|=\left|e^{\frac{-(\sqrt{\Delta x^{2}+\Delta y^{2}}{)}^{2}}{d^{2}}}-1\right|<0.05$$

specifically, in Equation (15):

(16) $$0.95<e^{\frac{-(\sqrt{\Delta x^{2}+\Delta y^{2}}{)}^{2}}{d^{2}}}<1.05$$

and in Equation (16):

(17) $$\Delta x^{2}+\Delta y^{2}<0.05d^{2}$$

where *d*_i_ is the diameter of the Gaussian function *m*(*r*_s_), as shown in Figure 3b:

(18) $$d_{i}=\frac{6}{\sqrt{2}}d$$

according to Equation (18), the nozzle positioning errors (Δ*x*, Δ*y*) are as follows:

(19) $$\Delta x<0.037\cdot d_{i}$$

(20) $$\Delta y<0.037\cdot d_{i}$$

Deterministic polishing based on Jet-ECM is a special stress-free polishing that has a Gaussian-type MRR function. The nozzle positioning errors are related to the diameter of the MRR profile. A larger diameter of the MRR can lower the nozzle positioning accuracy requirements. With the decrease in the MRR diameter, the requirements of the nozzle positioning accuracy are increased. According to the existing experimental results [24], *d*_i_ is 3 mm. It can be seen from Equations (19) and (20) that the positioning errors of the nozzle in the X and Y directions must be less than 110 μm, respectively. The nozzle positioning errors include device errors and installation errors. According to the principle of error distribution [28], the device error should be less than 27 μm.

## 3. Experiments

### 3.1. Experimental Device Design

Based on the model of Section 2.3, a specific stress-free deterministic polishing system was designed. The whole system can be divided into three parts: mechanical devices, motion control systems, and accessory devices. The mechanical devices include X, Y, Z rails and motors. The workpiece is placed in an electrolytic cell, which can move along the Y-rail. The nozzle is fixed on the force sensor, which can move along the X-rail and Z-rail. The relative movements between the workpiece and nozzle are realized using the motion control systems. The accessory devices include a gap control system and an electrolyte supply system.

#### 3.1.1. Mechanical Devices

**Figure 4:**
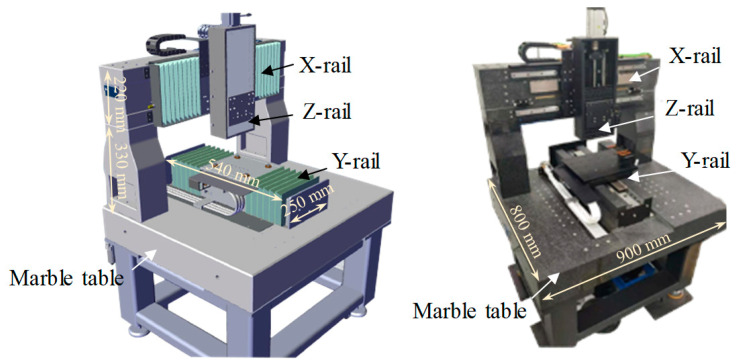
The mechanical device of deterministic polishing.

The moving parts of the mechanical devices include X, Y, and Z rails (Figure 4). The working lengths of the three rails are 250 mm, 300 mm, and 200 mm, respectively. The relative movements are achieved through the linkage control of the three rails. The electrolytic cell and the leveling devices are placed on the Y-rail. The Y-rail adopts an air-floating rail to avoid the crawling phenomenon caused by friction. The X and Y rails are driven by motors. The Z-rail uses a servo motor with a brake and a high-precision ball screw. The base of the mechanical device is made of marble, which can keep the mechanical device maintaining good accuracy.

#### 3.1.2. Motion Control System

The motion control system adopts a closed-loop control system. The motion control system includes a motion controller, motors, and measuring devices. The flowchart of the process is as follows. First, the motion instructions are transported to the motion controller. Then, the controller drives the rail to the corresponding position according to the instructions. The measuring device measures the actual position of the rail for correction of the positioning errors. The programmable multi-axis controller (PMAC, Delta Tau Data Systems, Inc., Chatsworth, CA, USA) is used as the motion controller. The PMAC controller is used in conjunction with high-performance motors and gratings to achieve the high precision movements of the rails. In addition, based on Power PMAC IDE (4.3.2.19) and Microsoft Visual Studio (Microsoft Visual Studio Community 2017), the motion control software was developed. The motion control software includes manual control function, automatic control function, coordinate display function, zero-back function, and G code function. The zero-back function is to return all the rails to the initial positions.

#### 3.1.3. Accessory Devices

**Figure 5:**
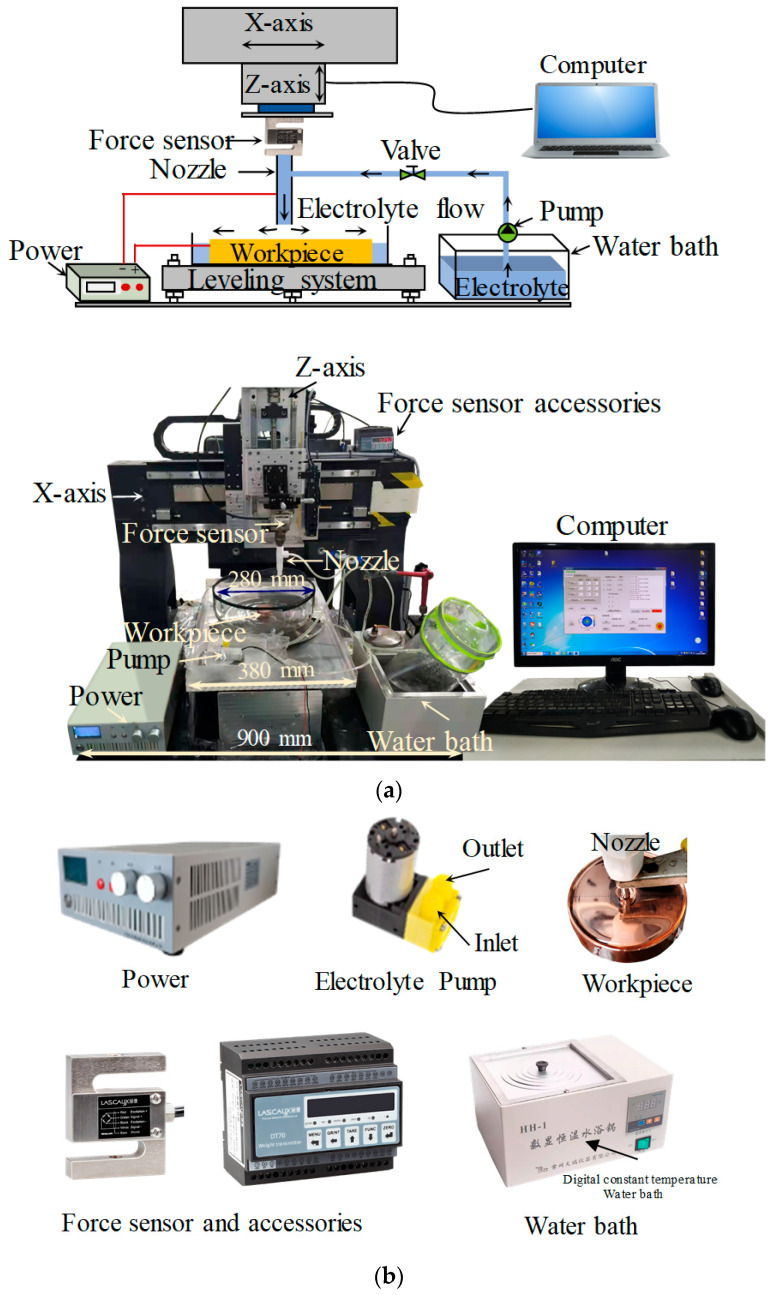
The deterministic polishing system. (**a**) Schematic diagram and actual pictures of the polishing system. (**b**) Detailed pictures of the device.

The accessory devices include a leveling system, a gap control system, and an electrolyte supply system. The leveling system is used to ensure the horizontal of the workpiece. The force sensor is used to judge the contact state between the nozzle and the workpiece. When the nozzle contacts the workpiece, the force sensor can detect the contact force. This nozzle position would be defined as the zero position. Then, the nozzle can be moved far away from the zero position by a certain distance in the Z-direction to adjust the machining gap. The electrolyte supply system includes a water bath, two pumps, and an electrolyzer. The electrolyte is stored in the water bath to maintain the electrolyte temperature during processing. The electrolyte is circulated by two pumps. The specific stress-free deterministic polishing system is shown in Figure 5.

### 3.2. Experimental Parameters

A cylindrical nickel nozzle with an inner diameter of 1 mm was used in the experiments, which has excellent electrical conductivity, good corrosion resistance, and suitable tensile strength. Pure Cu was used as the workpiece. Phosphate-based acidic electrolyte with high viscosity was used to avoid the generation of flocculent precipitates to improve the processing stability [29].

A detailed composition of the electrolyte is shown in Table 1. The temperature of the electrolyte was maintained at 35 °C. The electrolyte is shot from the nozzle to the workpiece at a certain flow rate to form a stable hydraulic jump.

The power (Suzhou Nine-dream Information Technology Co., Ltd., Suzhou, China) was used to provide power. Also, the duty cycle (0–100%) can be adjusted. The detailed processing parameters are shown in Table 2. A laser confocal microscope (VK-X250, Keyence Co., Osaka, Japan) was used to measure the profiles of the pits. The Cu surface flatness was measured using the flatness measuring instrument (FlatMaster200, Corning Incorporated, Corning, NY, USA) with a measuring range of *φ* = 45 mm.

## 4. Results and Discussion

### 4.1. The Positioning Accuracy of the Assembled Device

After the polishing device is assembled, the positioning accuracy of the motion system needs to be measured. The laser interferometer (XL80, Renishaw, West Dundee, IL, USA) was used to measure the positioning error of the X and Y rails. The XL80 laser interferometer system includes a laser device, a reflector, a spectroscope, and the Renishaw calibration system data analysis software (Version 20.02.02).

**Figure 6:**
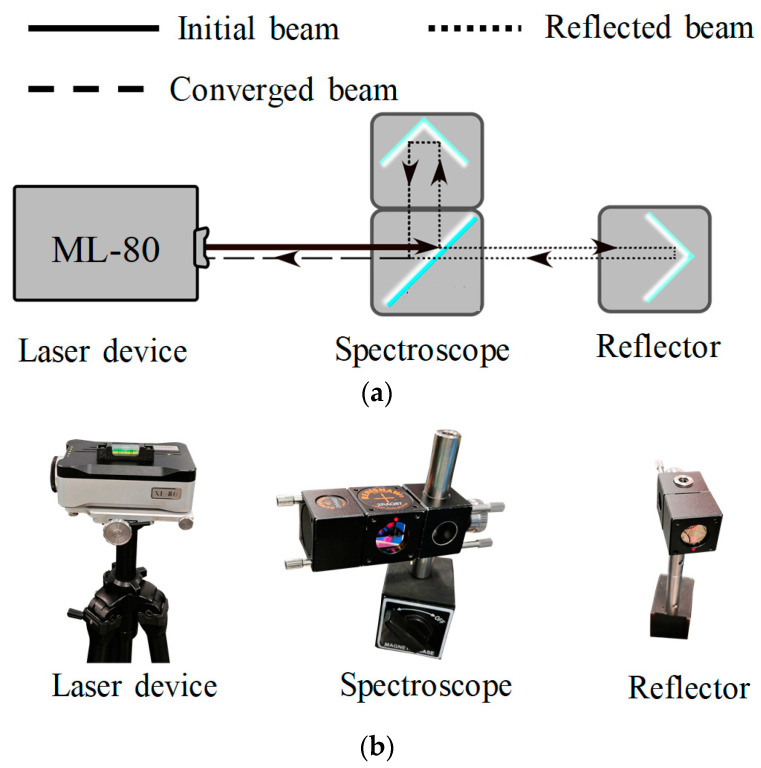
The schematic of measurement (**a**) Schematic diagram; (**b**) ML80 measuring devices.

During the measurement, the laser device emits a laser, which is divided into two beams. One of the beams is reflected using a fixed reflector, which keeps stationary during the measurement. Another beam is reflected using the spectroscope, which is attached to the moving part of the rail during the measurement. The two beams are converged and monitored using the XL80 device. By recording the changes in the interference pattern, the movement distance of the moving part can be calculated (Figure 6).

**Figure 7:**
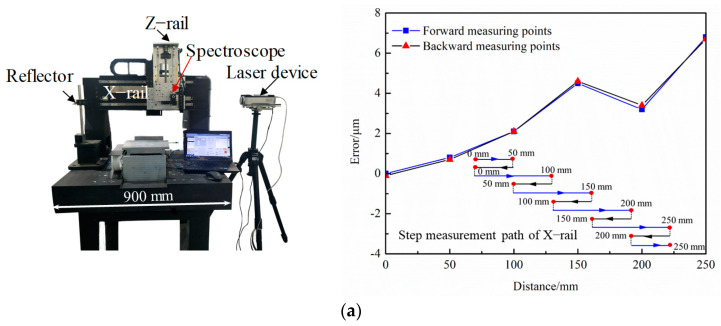

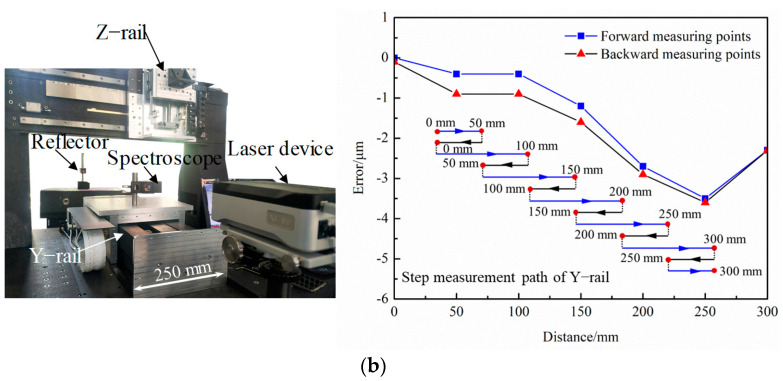
The process of the measurements. (**a**) Measurement of X-rail. (**b**) Measurement of Y-rail (The blue arrows represent the forward direction, the black arrows represent the backward direction, and the dashed lines represent no movement).

As is shown in Figure 7, the reflector is fixed on the marble platform. The spectroscope is attached to the moving part of the testing rail. During the test, the X-rail with a length of 250 mm is divided into segments. A step measurement method is adopted, which makes the trajectory look snake-shaped. First, the moving part moved from the initial position (0 mm position) of the rail to the 50 mm position and the measurement data were collected at 50 mm position. After that, the moving part returned back to the initial position (0 mm), and the measurement data were collected. Then the moving part moved from the initial position (0 mm) to the 100 mm position and the measurement data were collected at the 100 mm position. Then, the moving part moved back to the 50 mm position, and the measurement data were collected. The process was repeated, which made the moving part trajectory just look snake-shaped until the moving part arrived at the end of the X-rail (250 mm position).

During the X-rail test process, twelve measurement data were collected. After testing, the Renishaw analysis software (Renishaw Company, West Dundee, IL, USA) was used to evaluate the data measured using the laser interferometer. The measurement results show that the positioning error of the X-rail is 6.9 μm and the repeated positioning error is 0.2 μm. The positioning error of the Y-rail is 3.6 μm, and the repeated positioning error is 0.5 μm. Based on the model of Section 2.3, the accuracy of the motion system meets the requirements.

### 4.2. The MRR Function of Jet-ECM

To measure the MRR function, the most commonly used method is the spot method. The spot method uses a microscope to measure the 3D profile of the pit to calculate the total material removal amounts. Based on the material removal amounts and the processing time, the MRR can be calculated [30]. The process is detailed. The first step is to process a pit on the workpiece using Jet-ECM. The cross-sectional profile of the pit is axis-symmetrical Gaussian-type. Use MATLAB software Curve Fitting APP (Version R2018b) to fit the profile function of the pit. Based on the fitted function of the cross-sectional profile and the processing time, the MRR function can be calculated.

**Figure 8:**
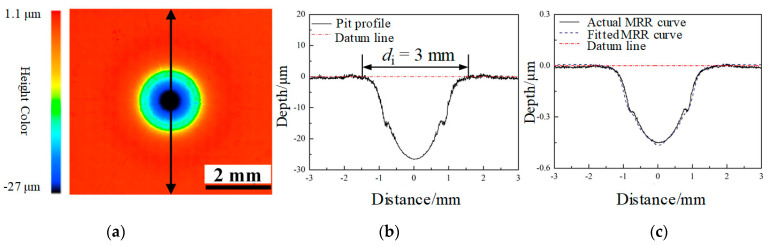
MRR function fitting of the Jet-ECM. (**a**) The spot obtained after Jet-ECM. (**b**) The cross-sectional profile of the pit. (**c**) The actual profile and the fitted MRR function profile.

As is shown in Figure 8a, the pit can be measured using the laser confocal microscope after processing for 60 s using the parameters in Table 2. As displayed in Figure 8b, the two-dimensional cross-sectional profile of the spot is obtained. The cross-sectional profile of the pit is axis-symmetrical, and the profile range is constrained to an area of ±1.5 mm and the pit depth is 26.5 μm. The depth of the material removal decreased significantly since the distance was far away from the symmetry axis. The profile function could be fitted with the MATLAB software Curve Fitting APP (Version R2018b) to obtain the cross-sectional profile function. The MRR function can be obtained according to the profile function and the processing time.

(21) $$m(r_{z})=-1.41\times e^{{\left(-\frac{r_{z}}{0.8}\right)}^{2}}-0.3\times e^{{\left(-\frac{\left(r_{z}-0.9\right)}{0.1669}\right)}^{2}}-0.3\times e^{{\left(-\frac{\left(r_{z}+0.9\right)}{0.1669}\right)}^{2}}-0.23\times e^{{\left(-\frac{\left(r_{z}-0.6\right)}{0.23}\right)}^{2}}-0.23\times e^{{\left(-\frac{\left(r_{z}+0.6\right)}{0.23}\right)}^{2}}$$

where *r*_z_ is the distance from a random point to the symmetry axis.

To verify the fitted MRR function, the actual profile and the MRR function fitting profile were compared. The deviation between the actual profile (solid line) and the MRR function fitting profile (dash line) is nanoscale (Figure 8c).

### 4.3. The Polishing Results of the Stress-Free Deterministic Polishing Based on Jet-ECM

**Figure 9:**
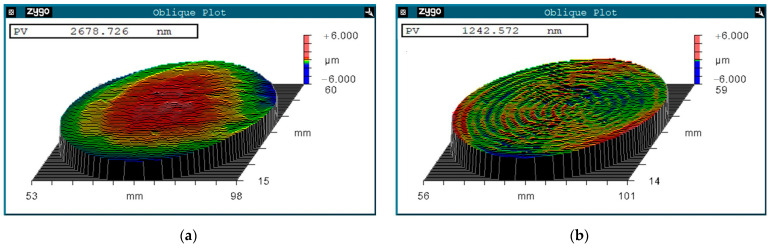
Topographic of the Cu workpiece (**a**) Initial PV = 2.67 μm; (**b**) After process PV = 1.24 μm.

The initial morphology of the workpiece was measured, as shown in Figure 9a. The surface PV value of the *φ* 50 mm workpiece (valid dimensions = 90% of the central region) before polishing is 2.67 μm. Combined with the MRR and the theoretical model [18,19], detailed processing parameters, such as nozzle traveling path (*e*_i_, *n*_i_) and moving speeds *ω*_i_ are shown in Table 3.

According to the processing parameters in Table 3. The processing time is 34 min. After polishing, the surface PV value of the *φ* 50 mm workpiece (valid dimensions = 90% of the central region) converged from 2.67 μm to 1.24 μm. The bump at the center of the workpiece (red color) is completely removed, achieving a flat surface (Figure 9b).

**Figure 10:**
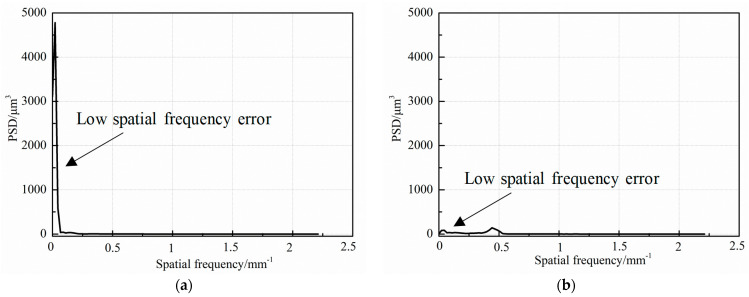
Low spatial frequency error analysis (**a**) Before process; (**b**) After process.

To achieve clearer and more intuitively displayed results for assessing the height distribution and characteristics of the workpiece surface (Figure 9), the power spectral density (PSD) method is applied, which can display the height distribution and characteristics of the surface more clearly and intuitively [31]. The spatial frequency from low to high corresponds to flatness, waveness, and roughness. To analyze the low frequency spatial errors of the workpiece, twenty diameters in Figure 9 at equal angles were chosen for PSD calculation. The calculated PSD data were averaged for a more accurate assessment. As shown in Figure 10a, before the specific stress-free deterministic polishing, the low frequency spatial errors of the workpiece surface are significant. After the stress-free deterministic polishing, most of the low frequency spatial errors are eliminated, which means the surface flatness is significantly improved, as shown in Figure 10b.

## 5. Conclusions

The stress-free deterministic polishing based on Jet-ECM was used to process an ultra-flat Cu surface. A mathematical model was established to analyze the impact of nozzle positioning errors on specific stress-free deterministic polishing. Based on the model, machine devices were designed, and an ultra-flat Cu surface was achieved. The conclusions could be concluded.

(1) The stress-free deterministic polishing based on Jet-ECM combines electrochemical dissolution, nozzle path planning, and nozzle speed control to achieve the flattening of the workpiece. Through this method, the PV value of the surface can be effectively reduced, which indicates that surface shape accuracy was improved. Also, it can provide an important supplement to the deterministic processing.
(2) When the actual nozzle position deviates from the predicted position, the material removal amounts distribution of the workpiece is changed, resulting in machining errors. In Jet-ECM, the MRR is Gaussian-type. It can be concluded from the mathematical model that the requirements of the machine device accuracy are related to the shape of the material removal. The larger the MRR diameter *d*_i_, the lower the machine device accuracy requirement is needed. As the diameter of the MRR decreases, the requirements for positioning accuracy increase. When the *d*_i_ is 3 mm, the nozzle positioning errors in the X and Y directions are required to be less than 110 μm. Based on the principle of precision distribution, the positioning accuracy of the machine device should be superior to 27 μm, respectively.
(3) The specific device for the stress-free deterministic polishing based on Jet-ECM was designed. The positioning errors of the X-rail and Y-rail of the machine device are 6.9 μm and 3.6 μm. The specific device was used for stress-free deterministic polishing based on Jet-ECM. After processing for 34 min, the PV value of the surface was reduced from 2.67 μm to 1.24 μm. According to the PSD analysis, the low frequency spatial errors of the surface height distribution were significantly eliminated after polishing, which means the surface shape accuracy was significantly improved.

## Figures and Tables

**Table 1:** Composition of the phosphoric acid-based electrolyte.

Composition	Value
Phosphoric acid/mL	425
Ethanol/mL	45
Lactic acid/mL	30
Benzotriazole/g	3
Ammonium acetate/g	1.5

**Table 2:** Processing parameters of Jet-ECM.

Parameters	Value
Voltage/V	6.5
Frequency/kHz	10
Duty ratio/%	50
Gap/mm	0.6
Electrolyte temperature/°C	35

**Table 3:** Parameters of deterministic polishing.

Ring Groove Serial Numbers	Revolution Radius *e*_i_ (mm)	Nozzle Moving Speed *ω*_i_ (rad/s)	Revolution Numbers *n*_i_
1	*e*_1_ = 1	4π/12	2
2	*e*_2_ = 2	4π/18	2
3	*e*_3_ = 3	4π/35	2
4	*e*_4_ = 4	4π/35	2
5	*e*_5_ = 5	4π/59	2
6	*e*_6_ = 6	4π/47	2
7	*e*_7_ = 7	4π/85	2
8	*e*_8_ = 8	4π/60	2
9	*e*_9_ = 9	4π/105	2
10	*e*_10_ = 10	4π/75	2
11	*e*_11_ = 11	4π/115	2
12	*e*_12_ = 12	4π/90	2
13	*e*_13_ = 13	4π/130	2
14	*e*_14_ = 14	4π/95	2
15	*e*_15_ = 15	4π/140	2
16	*e*_16_ = 16	4π/95	2
17	*e*_17_ = 17	4π/145	2
18	*e*_18_ = 18	4π/100	2
19	*e*_19_ = 19	4π/140	2
20	*e*_20_ = 20	4π/85	2
21	*e*_21_ = 21	4π/135	2
22	*e*_22_ = 22	4π/80	2
23	*e*_23_ = 23	4π/120	2
24	*e*_24_ = 24	4π/45	2

*e*_i_ is the distance between the nozzle center and the workpiece symmetric center.

## Data Availability

Data are contained within the article.

## Nomenclature

**Symbol**	**Explanation**
*r*_s_	The distance between an arbitrary predicted nozzle position A and a random location B on the workpiece surface
*r*_s_′	The distance between the actual nozzle position A′ and a random location B on the workpiece surface
*m*(*r*_s_)	A mathematical function to describe the line profile of the Jet-ECM MRR
Δ*x*	Nozzle positioning error in X direction
Δ*y*	Nozzle positioning error in Y direction
*ε*	The deviation between the theoretical MRR and actual MRR
*k*_w_	The deviation rate
*d*	The eigenvalue of the Gaussian function
*d*_i_	The diameter of the pit machined by Jet-ECM
*r*_z_	The distance from a random point to the symmetry axis
*e*_i_	The distance between the nozzle center and the workpiece symmetric center which represents the nozzle revolution radius during the deterministic polishing
*ω*_i_	Nozzle moving speed during the deterministic polishing
*n*_i_	Nozzle revolution numbers in *e*_i_
PV	The surface peak and valley value
CMP	Chemical mechanical polishing
CCOS	Computer-controlled optical surfacing
Jet-ECM	Jet electrochemical machining
MRR	The material removal rate
PSD	The power spectral density
